# Rare Sequence Variation Underlying Suspected Familial Cerebral Small‐Vessel Disease

**DOI:** 10.1161/JAHA.123.035771

**Published:** 2024-07-31

**Authors:** Bernard P. H. Cho, Kate Auckland, Stefan Gräf, Hugh S. Markus

**Affiliations:** ^1^ Stroke Research Group Department of Clinical Neurosciences University of Cambridge Cambridge UK; ^2^ Department of Medicine University of Cambridge Victor Phillip Dahdaleh Heart and Lung Research Institute Cambridge UK

**Keywords:** burden test, familial stroke, National Institute for Health Research BioResource Rare Disease study, whole‐genome sequencing, Genetic, Association Studies, Epidemiology, Precision Medicine, CADASIL, Cerebrovascular Disease/Stroke

## Abstract

**Background:**

Cerebral small‐vessel disease (cSVD) is the leading monogenic cause of stroke. Despite genetic screening in routine diagnosis, many cases remain without a known causative variant. Using a cohort with suspected familial cSVD and whole‐genome sequencing, we screened for variants in genes associated with monogenic cSVD and searched for novel variants associated with the disease.

**Methods and Results:**

Rare variants were identified in whole‐genome sequencing data from the NBR (National Institute for Health Research BioResource Rare Disease) study. Pathogenic variants in known monogenic cSVD genes were identified. Gene‐based burden tests and family analysis were performed to identify novel variants associated with familial cSVD. A total of 257 suspected cSVD cases (mean ± SD age, 56.2 ± 16.1 years), and 13 086 controls with other nonstroke diseases (5874 [44.9%] men) were studied. A total of 8.9% of the cases carried a variant in known cSVD genes. Excluding these known causes, 23.6% of unrelated subjects with cSVD carried predicted deleterious variants in the Genomics England gene panel, but no association was found with cSVD in burden tests. We identified potential associations with cSVD in noncoding genes, including *RP4‐568F9.3* (adjusted *P* = 7.1 × 10^−25^), *RP3‐466I7.1* (adjusted *P* = 8.9 × 10^−16^), and *ZNF209P* (adjusted *P* = 1.0 × 10^−15^), and matrisomal genes (adjusted *P* = 5.1 × 10^−6^), including *FAM20C*, *INHA*, *LAMC1*, and *VWA5B2*.

**Conclusions:**

Predicted deleterious variants in known cSVD genes were present in 23.6% of unrelated cases with cSVD, but none of the genes were associated with the disease. Rare variants in noncoding and matrisomal genes could potentially contribute to cSVD development. These genes could play a role in tissue development and brain endothelial cell function. However, further studies are needed to confirm their pathophysiological roles.

**Nonstandard Abbreviations and Acronyms**
BeviMed: Bayesian evaluation of variant involvement in Mendelian disease
CADASIL: cerebral autosomal dominant arteriopathy with subcortical infarcts and leukoencephalopathy
CADD: combined annotation‐dependent depletion
cSVD: cerebral small‐vessel disease
MAF: minor allele frequency
NBR: National Institute for Health Research BioResource Rare Disease
SKAT‐O: sequence kernel association test with optimal combination of variants
WGS: whole‐genome sequencing

**Clinical Perspective**

**What Is New?**

- Many patients with apparent familial cerebral small‐vessel disease have unknown causative variants despite routine genetic screening; this study used whole‐genome sequencing in a large cohort of well‐phenotyped suspected familial cerebral small‐vessel disease to address this challenge.
- Rare sequence variants in noncoding and matrisomal genes, including *FAM20C*, *INHA*, *LAMC1*, *RP3‐466I7.1*, *RP4‐568F9.3*, *VWA5B2*, and *ZNF209P*, were associated with cerebral small‐vessel disease.

**What Are the Clinical Implications?**

- These novel variants could play a role in tissue development and brain endothelial cell function, but further studies are needed to confirm their pathophysiological roles and potential as therapeutic targets.

The most common subtype of stroke caused by monogenic disease is cerebral small‐vessel disease (cSVD), which results in small deep lacunar infarcts and more diffuse chronic white matter changes seen as white matter hyperintensities on magnetic resonance imaging (MRI).^1^ The discovery of genes for several types of monogenic cSVD has provided important insights into their underlying mechanisms.^2^, ^3^, ^4^, ^5^, ^6^ The most common single‐gene disorders identified to cause cSVD are cerebral autosomal dominant arteriopathy with subcortical infarcts and leukoencephalopathy (CADASIL) attributable to *NOTCH3* mutations, CADASIL2 attributable to autosomal dominant *HTRA1* mutations, and autosomal dominant *COL4A1/2* disease.^2^ In addition, several rarer causes have been recently reported, including *ARHGEF15*,^3^ *ATP11B*,^4^ *CECR1*,^5^ *CSF1R*,^6^ *PLOD3*,^7^ and *LAMB1*.^8^ Many of these genes encode proteins in the cerebrovascular matrisome, suggesting perturbations in this ensemble of extracellular matrix proteins may be a convergent mechanism in cSVD.^9^, ^10^ However, despite screening for all known monogenic cSVD genes in routine diagnosis, <15% of adult patients with apparent monogenic cSVD had a causative variant identified,^11^ suggesting there are many yet unidentified monogenic causes of cSVD. Thus, the identification of new causative genes is necessary to improve diagnosis in individual patients. Furthermore, this may also reveal novel disease mechanisms for cSVD, and potentially new treatment approaches.

Identifying additional genes underlying monogenic cSVD requires whole‐exome or whole‐genome sequencing (WGS) in well‐characterized cohorts with the clinical phenotype of familial cSVD. However, there are few such resources available. As part of the 100 000 Genomes Project pilot, patients with suspected monogenic cSVD, their relatives, and patients with other rare diseases were recruited for WGS to the NBR (National Institute for Health Research BioResource Rare Disease) study.^12^ The patients with suspected monogenic cSVD had a typical clinical and neuroradiological phenotype of the disease, and some families showed a Mendelian pattern of inheritance, but routine clinical genetic testing in the National Health Service, primarily for *NOTCH3*, had been negative. This unique data set provides the potential to answer several important research questions. First, how common are rare variants in known familial cSVD genes among patients presenting with suspected familial cSVD, and second, can we identify novel genes underlying cSVD?

Therefore, using data from suspected cases of monogenic cSVD in the NBR study, we assessed the prevalence of rare variants in previously identified forms of monogenic cSVD, and applied a variety of bioinformatic techniques to identify novel rare variants that may cause monogenic cSVD.

## Methods

All data and materials from the NBR study are available to bona fide researchers on application (https://bioresource.nihr.ac.uk/using‐our‐bioresource/apply‐for‐bioresource‐data‐access/). This study was performed under project code RDC‐CSV.

### Study Population

**Figure 1:**
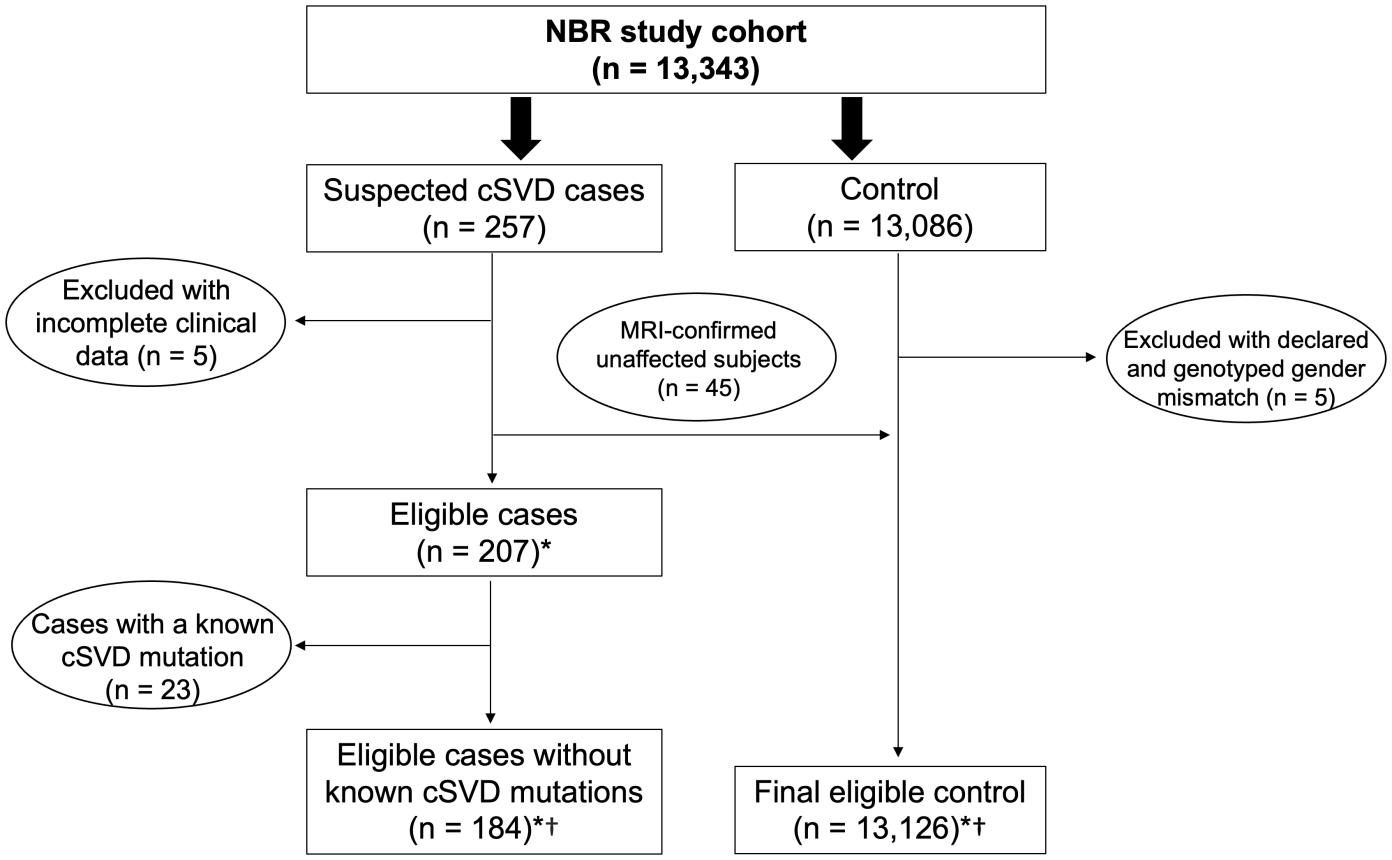
Flowchart showing the screening of cases and controls. Cases are defined as subjects with magnetic resonance imaging (MRI)–confirmed features of cerebral small‐vessel disease (cSVD; evidence beyond what is expected from age and risk factors), stroke, or vascular dementia (n=161). Cases also include the relatives of patients who have other cause of stroke or may not have a brain MRI/computed tomography scan (n=46); notably, these related cases were included in the genome‐wide sequence kernel association test with optimal combination of variants (SKAT‐O) and excluded from the subsequent candidate gene burden tests. Controls are defined as subjects who were confirmed to be without stroke. *Denotes subjects were included in candidate gene burden tests. †Denotes subjects were included in the genome‐wide SKAT‐O. NBR indicates National Institute for Health Research BioResource Rare Disease.

The NBR study, a pilot of the Genomics England 100 000 Genomes Project, recruited and sequenced the whole genomes of individuals across 15 rare disease domains.^12^ From October 2014 to October 2016, patients with a suspected familial cSVD, who had tested negative for any known monogenic cSVD mutation in routine clinically available testing provided by the National Health Service, were recruited to the study. Whole‐genome sequences of 13 343 participants were analyzed, 257 of which were individuals recruited under the cSVD domain (Figure 1). Suspected familial cSVD (N=161) was defined as young onset (usually before the age of 60 years) lacunar stroke or vascular dementia, and MRI features of cSVD (white matter hyperintensities, lacunes, or both). Once an index case was identified, we also recruited affected and unaffected relatives where available. Unaffected family members (N=45) were defined as individuals aged ≥30 years who did not have features of cSVD on MRI, and had no history of stroke or cognitive impairment. In some cases (N=46), family members were classified as unknown status because of an absence of computed tomography or brain imaging to assess cSVD or another stroke cause other than cSVD (eg, cardioembolic or large‐artery strokes). Five family members could not be classified because of inadequate information available.

The non‐cSVD control cohort (n=13 131) consisted of subjects with confirmed unaffected status (0.3%), bleeding, thrombotic, and platelet disorders (9.4%), pulmonary arterial hypertension (9.2%), Ehlers‐Danlos syndrome (0.2%), Genomics England Ltd project participants (35.8%), hypertrophic cardiomyopathy (2.0%), intrahepatic cholestasis of pregnancy (2.3%), Leber hereditary optic neuropathy (0.5%), multiple primary tumors (4.5%), neuropathic pain disorder (1.6%), primary immune disorders (10.8%), primary membranoproliferative glomerulonephritis (1.4%), stem cell and myeloid disorders (2.0%), corticosteroid‐resistant nephrotic syndrome (2.1%), and others (17.9%). Prespecified enrollment criteria for the groups in the control cohort were previously described.^12^ None had a history of stroke or dementia. Five individuals with a discrepancy between genotyped and declared gender were excluded (which brought the total number of controls to 13 126) as such discrepancies may arise from errors in sample processing or could indicate a variance between declared gender and biological sex.

The NBR study received ethical approval from the East of England Cambridge South National Research Ethics Committee (reference: 13/EE/0325) and appropriate national ethics authorities in overseas enrollment centers. Written informed consent was obtained from all anonymized participants.

### Whole‐Genome Sequencing

Details about the bioinformatics analysis of WGS were previously described.^12^ Briefly, DNA was extracted from the venous blood samples, and WGS was performed by Illumina. The DNA samples were fragmented into ≈450 base pairs and processed using the Illumina TruSeq DNA PCR‐Free Sample Preparation kit. These were sequenced in batches at read lengths of 100, 125, and 150 base pairs. Samples with 100 and 125 base‐pair reads were sequenced on Illumina HiSeq 2500, whereas samples with 150 base‐pair reads were on a HiSeq X sequencer. Only samples with a minimum coverage of 15× for at least 95% of the genome passed quality control, with a mean coverage of 35×. The sequencing reads were then mapped to the GRCh37 reference genome by Illumina using the Isaac Aligner and Variant Caller, version 2 (Illumina Inc).

### Variant Format and Annotation

Variants identified from WGS data were normalized and merged into multisample VCF files by chromosome. They were annotated using Ensembl Variant Effect Predictor^13^ to include in silico variant pathogenicity scores, such as combined annotation‐dependent depletion (CADD),^14^ sorting intolerant from tolerant,^15^ and PolyPhen‐2,^16^ which were used for variant filtering.

### Estimation of Ethnicity and Relatedness

Population structure and relatedness were inferred on the basis of a representative set of single‐nucleotide polymorphisms using the R package GENESIS to perform PC‐AiR^17^ and PC‐Relate,^18^ respectively. The 35 114 autosomal single‐nucleotide polymorphisms selected from the Illumina genotyping arrays did not overlap quality control excluded regions or multiallelic sites in the 1000 Genomes phase 3 data set^19^ and had a minor allele frequency (MAF) of ≥0.3. To remove single‐nucleotide polymorphisms that are in strong linkage disequilibrium with each other, linkage disequilibrium pruning was performed using PLINK^20^ with a window size of 50 bp, a window shift of 5 bp, and a variance inflation factor threshold of 2. A total of 2110 samples from the 1000 Genomes Project (an external control database), which included European, African, South Asian, and East Asian populations, were filtered for the selected single‐nucleotide polymorphisms. These filtered samples were used to perform a principal component analysis using PC‐AiR. Genotypes from the NBR study samples were mapped to the loadings for the leading 5 principal components from the 1000 Genomes principal component analysis, and the likelihood that each sample belonged to each subpopulation was computed. Each sample was allocated to the population with the highest likelihood unless the likelihood was similar to other populations, in which case it was labeled as “other.” Additionally, related individuals were identified using the first 20 PCs from PC‐AiR, where pairwise identity‐by‐state distances and kinship values were extracted. The pairwise information was used by Primus to infer family networks and calculate the maximum set of unrelated samples.

Of the 13 343 participants, 82.0% were assigned to non‐Finnish European (n=10 940), 7.2% to South Asian (n=956), 2.1% to African (n=285), 0.7% to East Asian (n=89), 0.1% to Finnish‐European (n=18), and 7.9% to other (n=1055). A total of 10 510 unrelated individuals were retrieved, representing 78.8% of the study cohort.

### Cohort Definition and Allele Frequency Calculation

On the basis of the relatedness analysis, we defined the following sample subsets: (a) the maximum number of unrelated non‐cSVD controls (n=10 384, including those labeled as unaffected in Figure 1), (b) all participants recruited under the cSVD domain (n=212), and (c) all unrelated cSVD index cases (n=126). These subsets were used to annotate the variants in the multisample VCF file with calculated MAF using the fill‐tags extension of BCFtools.^21^

### Identification of Genes Causing cSVD

We followed the following bioinformatic pipeline:

1. Identify pathogenic variants in already known monogenic cSVD genes.
2. Perform a burden (Fisher exact) test and Bayesian evaluation of variant involvement in Mendelian disease (BeviMed) of predicted deleterious rare variants in previously reported monogenic cSVD genes included in the Genomics England panel (n=14).
3. Perform a burden test and BeviMed on rare variants in recently described monogenic cSVD genes that are not yet in clinical testing panels.
4. Perform sequence kernel association test with optimal combination of variants (SKAT‐O),^22^ followed by a family‐based analysis to identify novel genes and variants associated with monogenic cSVD.

### Selection of Candidate Genes

A list of 14 protein‐coding genes associated with cSVD was curated from the Genomics England gene panel for familial cSVD, version 1.16 (https://panelapp.genomicsengland.co.uk/panels/50/) for the initial candidate gene analysis. These genes include *ABCC6*, *APP*, *ATP1A2*, *CACNA1A*, *COL3A1*, *COL4A1*, *COL4A2*, *COLGALT1*, *CTSA*, *FOXC1*, *GLA*, *HTRA1*, *NOTCH3*, and *TREX1*. Additionally, a literature search was conducted to identify recently implicated cSVD genes, and 6 further genes were identified: *ARHGEF15*,^3^ *ATP11B*,^4^ *CECR1*,^5^ *CSF1R*,^6^ *PLOD3*,^7^ and *LAMB1*.^8^

### Rare Variant Filtering

Different variant filtering strategies were used before candidate‐gene analyses and genome‐wide association of variants with cSVD (Figure S1). For the former, only variants that fulfilled the following criteria were included: (1) an MAF of <1 in 1000 unrelated controls; (2) a CADD score of ≥15, as recommended on the CADD website (https://cadd.gs.washington.edu/info); (3) protein‐truncating or missense variants in canonical transcripts present in subjects with cSVD; and (4) missense variants not predicted as both tolerated and benign by sorting intolerant from tolerant and PolyPhen‐2. For SKAT‐O, all protein‐truncating and missense variants with an MAF of <1 in 1000 unrelated controls were included. Notably, our computing system limitations prevented the calculation of CADD scores for all genome variants. As a result, we could only manually obtain CADD scores for variants in candidate genes on the CADD web app (https://cadd.gs.washington.edu/score). This constraint led us to apply the CADD >15 filter only to variants in the candidate genes during the burden test, whereas it was not implemented in the genome‐wide SKAT‐O (Figure S1).

### Association of Rare Variants With cSVD

Filtered variants were grouped per gene and consequence type (predicted protein‐truncating and missense), and subjects with at least 1 variant were counted (no double counting) per group and tested for association with disease. A Fisher exact test (1 sided, greater) was applied, and *P*<0.05 was considered statistically significant. BeviMed (R package “BeviMed”) was used to compute the posterior probability of disease association for the likely mode of inheritance.^23^ Both tests were performed using R, version 4.0.3.

### Rare Variant Analysis Using SKAT‐O

To further examine the aggregate effect of rare variants in each protein‐coding gene (excluding the genes included in the burden analysis above) on cSVD, SKAT‐O analysis was performed. Like the burden analysis, explained cSVD cases were excluded from this analysis. SKAT‐O increases the statistical power to detect genetic associations by combining variance‐component and burden tests. Only protein‐truncating and missense variants with MAF < 0.001 were included (Figure S1). The analysis was performed in RvTests^24^ using default parameters and β (1,25) weights, with adjustment for rare disease, read length, sex, and the top 5 ethnicity principal components. Variants that were included in the analysis were limited to those found in the canonical transcript of the protein‐coding genes in GRCh37 genome assembly. The Bonferroni correction for multiple testing was applied to determine the *P* values for genome‐wide significance (*P*<5×10^−8^). Genes with suggestive significance (*P*<5×10^−5^) that encode key matrisome proteins^10^ and are expressed in the brain^25^ were analyzed further with clinical information. Variants in these genes, which occurred in >1 affected subject, were selected for family‐based analysis.

## Results

### Identification of cSVD Cases With Known Mutations Causing cSVD

Five patients with cSVD were excluded because WGS data from their proband did not pass quality control, leaving 252 cases for analysis.

The most common mutations in genes already described to cause cSVD were *NOTCH3* causing CADASIL (ie, variants altering a cysteine residue in an epidermal growth factor‐like repeat of NOTCH3, 10 unique variants in 11 carriers), *HTRA1* (4 unique variants in 9 carriers), and *COL4A2* (1 splice variant in 2 carriers).^2^ Three of the *NOTCH3* variants were located in epidermal growth factor‐like repeats 1 to 6, and 5 were absent from population databases. Only 2 *HTRA1* variants affected the trypsin‐like protease domain, and all were previously reported in population cohorts^26^; notably, 3 were identified in the UK Biobank and patients with cSVD.^27^ The *COL4A2* variant was not previously described. Additionally, a recently reported pathogenic variant in *LAMB1* ^8^ was identified. Together, these mutations accounted for 8.9% of all cases, of which all were heterozygous, except for 1 homozygous *HTRA1* nonsense mutation carrier (Table 1). It is important to highlight that patients with known *NOTCH3* mutations were not recruited so the *NOTCH3* mutations detected had not been identified on previous screening, which had often been limited to screening of a limited number of exons. Therefore, our figure is likely to underestimate the frequency of *NOTCH3* mutations in an unselected cohort with suspected familial cSVD.

**Table 1:** List of Patients With cSVD With a Known Mutation (n=23)

Patient index	Previously reported mutation	Exon	Domain	Population cohort (frequency)
1	*HTRA1*: p.Pro331Leu	5	Trypsin	gnomAD (5)
2	*HTRA1*: p.Pro331Leu	5	Trypsin	gnomAD (5)
3	CADASIL: *NOTCH3*: p.Arg1143Cys	21	EGFR29	UK Biobank (133); gnomAD (142)
4	CADASIL: *NOTCH3*: p.Cys379Ser	7	EGFR9	Absent
5	*HTRA1*: p.Arg370Ter		PDZ	UK Biobank (24); gnomAD (34)
6	*HTRA1*: p.Arg370Ter		PDZ	UK Biobank (24); gnomAD (34)
7	*HTRA1*: p.Arg302Ter		Trypsin	gnomAD (18)
8	*HTRA1*: p.Arg302Ter (homozygous)		Trypsin	gnomAD (18)
9	*COL4A2* splice variant			
10	*COL4A2* splice variant			
11	*LAMB1*: p.Leu1730Ter	33		Absent
12	CADASIL: *NOTCH3*: p.Cys212Arg	4	EGFR5	Absent
13	CADASIL: *NOTCH3*: p.Arg169Cys	4	EGFR4	UK Biobank (2); gnomAD (3)
14	CADASIL: *NOTCH3*: p.Cys76Tyr	3	EGFR1	Absent
15	*HTRA1*: p.Val175Met	2		UK Biobank (4); gnomAD (6)
16	*HTRA1*: p.Val175Met	2		UK Biobank (4); gnomAD (6)
17	CADASIL: *NOTCH3*: p.Arg1231Cys	22	EGFR22	UK Biobank (255); DiscovEHR (84); gnomAD (772)
18	CADASIL: *NOTCH3*: p.Arg607Cys	11	EGFR15	UK Biobank (7); DiscovEHR (4); gnomAD (6)
19	CADASIL: *NOTCH3*: p.Arg607Cys	11	EGFR15	UK Biobank (7); DiscovEHR (4); gnomAD (6)
20	CADASIL: *NOTCH3*: p.Cys554Phe	11	EGFR14	UK Biobank (1); gnomAD (1)
21	CADASIL: *NOTCH3*: p.Cys388Arg	7	EGFR9	Absent
22	CADASIL: *NOTCH3*: p.Cys709Tyr	13	EGFR18	Absent
23	*HTRA1*: p.Arg370Ter		PDZ	UK Biobank (24), gnomAD (34)

Population frequencies were obtained from the UK Biobank,^27^ Geisinger DiscovEHR initiative cohort,^26^ and gnomAD, version 4. The mutations identified in the UK Biobank were also reported in patients with cSVD. CADASIL indicates cerebral autosomal dominant arteriopathy with subcortical infarcts and leukoencephalopathy; cSVD, cerebral small‐vessel disease; EGFR, epidermal growth factor‐like repeat; gnomAD, Genome Aggregation Database; and PDZ, Post‐synaptic density‐95, Discs large, Zona occludens‐1. DiscovEHR is a collaboration between the Regeneron Genetics Center and Geisinger Health Syste that couples high‐throughput sequencing to longitudinal electronic health records (EHRs).

**Figure 2:**
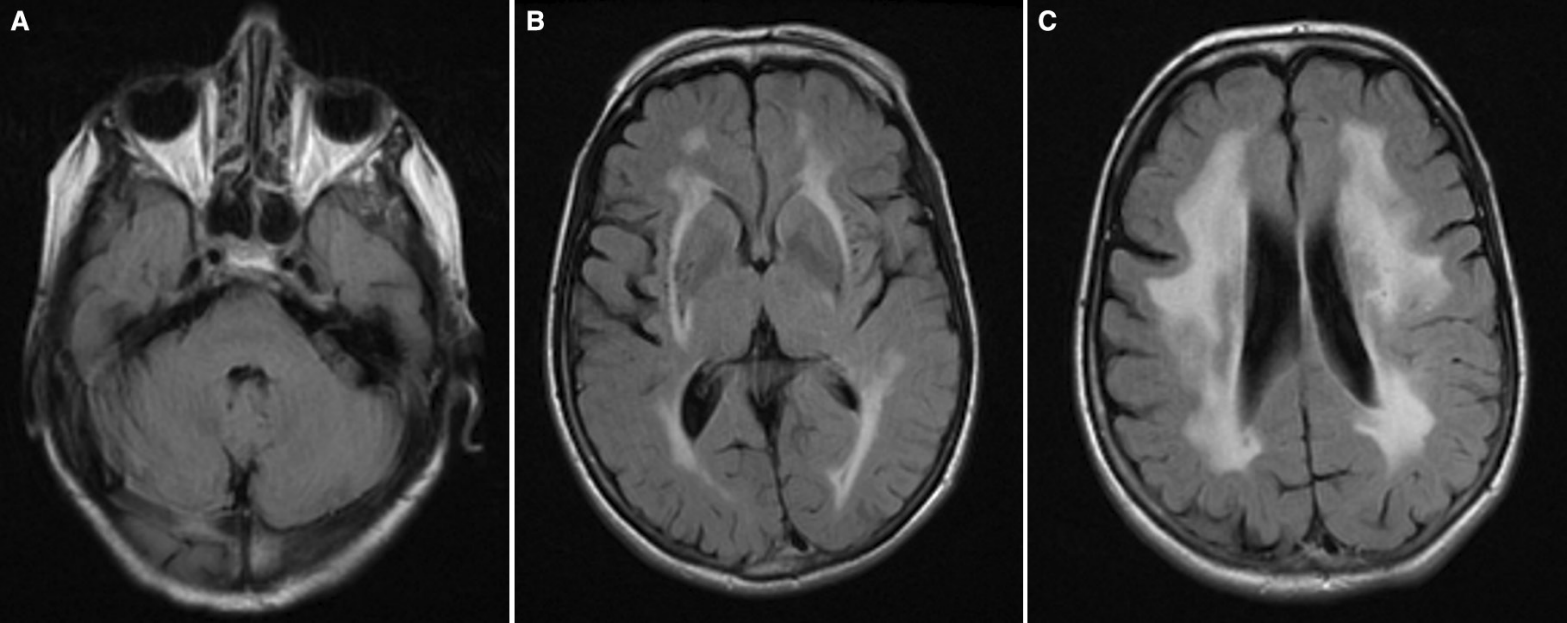
Axial fluid‐attenuated inversion recovery brain magnetic resonance imaging of a singleton case's (01‐007) pedigree. Carrier of *LAMB1*: p.Leu1730Ter variant, showing white matter hyperintensities in temporal lobes (**A**), external capsules (**B**), and around the ventricles (**C**).

The heterozygous carrier of the frameshift variant (ENSP00000222399.6: p.Leu1730Ter) in *LAMB1* was a female patient (01‐007, Figure S2). She had memory deficit and migraine with aura when she was recruited. She had transient ischemic attack at the age of 57 years, stroke at the age of 61 years, and passed away at the age of 67 years. Brain MRI taken at the age of 64 years showed extensive confluent white matter hyperintensities, and lacunar infarcts but no cerebral microbleeds. The white matter hyperintensities were widespread with prominent involvement of the external capsule but spared the anterior temporal poles (Figure 2). Although this patient was a singleton, she had a strong family history of stroke, with her father and 2 maternal aunts being affected. Additionally, the p.Leu1730Ter variant was found to segregate with an unrelated patient with cSVD in a French cohort, and functional analysis demonstrated that the resulting truncated proteins escaped nonsense‐mediated mRNA decay.^8^ Therefore, this variant was interpreted as likely pathogenic.

### Burden Test on Known Monogenic cSVD Genes

Of the 13 310 participants (5971 [44.9%] men, excluding the explained cases identified above), 229 were recruited to the cSVD domain. Following sample quality control, 106 unrelated cSVD cases (n=126, including those with a known mutation) and 10 380 unrelated controls were included in gene‐based burden tests and BeviMed analysis. After excluding the known cases of cSVD mutations, the associations previously observed between cSVD and *HTRA1* and *NOTCH3* were no longer present (Table 2).

**Table 2:** Fisher Exact Test Comparing the Burden of Rare Variants in Previously Reported cSVD Genes

All samples					Excluding known cSVD mutations			
Previously reported familial cSVD genes	Variant carriers in unrelated cases, n (%)	Variant carriers in unrelated controls, n (%)	Predicted mode of inheritance (posterior probability)	OR, *P* value	Variant carriers in unrelated cases, n (%)	Variant carriers in unrelated controls, n (%)	Predicted mode of inheritance (posterior probability)	OR, *P* value
*ABCC6*	9 (7.1)	1178 (11.3)	Dominant (0.99)	0.60, 0.96	8 (7.5)	1178 (11.3)	Dominant (0.99)	0.64, 0.92
*APP*	1 (0.8)	115 (1.1)	Unknown	0.71, 0.76	1 (0.9)	115 (1.1)	Unknown	0.85, 0.69
*ATP1A2*	0	35 (0.6)	Unknown	0, 1.00	0	35 (0.6)	Unknown	0, 1.00
*CACNA1A*	4 (3.2)	403 (3.9)	Dominant (1.00)	0.81, 0.73	3 (2.8)	403 (3.9)	Dominant (0.99)	0.72, 0.78
*COL3A1*	2 (1.6)	141 (1.4)	Dominant (0.97)	1.17, 0.51	2 (1.9)	141 (1.4)	Dominant (0.99)	1.40, 0.43
*COL4A1*	1 (0.8)	170 (1.6)	Unknown	0.48, 0.88	1 (0.9)	170 (1.6)	Unknown	0.57, 0.83
*COL4A2*	3 (2.4)	286 (2.8)	Dominant (0.89)	0.86, 0.68	3 (2.8)	286 (2.8)	Dominant (0.99)	1.03, 0.56
*COLGALT1*	1 (0.8)	129 (1.2)	Unknown	0.76, 0.74	1 (0.9)	129 (1.2)	Unknown	0.76, 0.74
*CTSA*	0	54 (0.5)	Unknown	0, 1.00	0	54 (0.5)	Unknown	0, 1.00
*FOXC1*	0	103 (1.2)	Unknown	0, 1.00	0	103 (1.2)	Unknown	0, 1.00
*GLA*	1 (0.8)	29 (0.3)	Unknown	2.85, 0.30	1 (0.9)	29 (0.3)	Unknown	3.40, 0.26
*HTRA1*	8 (6.3)	55 (0.5)	Dominant (0.98)	12.72, <0.001	2 (1.9)	55 (0.5)	Dominant (0.99)	3.61, 0.11
*NOTCH3*	12 (9.5)	303 (2.9)	Dominant (1.00)	3.50, <0.001	3 (2.8)	303 (2.9)	Dominant (0.99)	0.97, 0.60
*TREX1*	0	74 (0.7)	Unknown	0, 1.00	0	74 (0.7)	Unknown	0, 1.00

Comparison between unrelated cSVD cases with (n_1_=126) or without a known mutation (n_2_=106) vs unrelated controls (n_3_=10 380). cSVD indicates cerebral small‐vessel disease; and OR, odds ratio.

Without the explained cases, rare predicted deleterious variants in the Genomics England gene panel were found in 23.6% (25 of 106) of the unrelated subjects with cSVD. All the variants were heterozygous and predicted to be autosomal dominant by BeviMed. Twenty unique variants were identified across all of the genes, of which 7 were previously reported in dbSNP (Database for Single Nucleotide Polymorphisms and Other Classes of Minor Genetic Variation). Two of the variants (both in *ABCC6*) led to protein truncation, and the remainder were missense variants. Further details about the individual variants identified in cSVD cases are provided in Table S1.

Of the other genes in the Genomics England panel, rare predicted deleterious variants were identified in *ABCC6* (8 [7.5%] unrelated cases), *CACNA1A*, *COL4A2* (3 [2.8%] cases each), *COL3A1* (2 [1.9%] cases), and *APP*, *COL4A1*, *COLGALT1*, and *GLA* (1 [0.9%] case each). No variants were detected in *ATP1A2*, *CTSA*, *FOXC1*, or *TREX1* among cSVD cases.

### Recently Implicated cSVD Genes

Other recently implicated cSVD genes (n=7) were assessed for their association with the disease (Table 3). However, predicted deleterious variants were only found in *ARHGEF15*, *ATP11B*, *LAMB1*, and *PLOD3*, none of which were associated with cSVD. After excluding the known mutation cases, 7 unique variants were identified, all were missense variants, and 5 of them were reported in dbSNP (Table S2). Most of the variants had no evidence of segregation in families. A *PLOD3* variant (p.Glu660Lys) was carried by 2 related individuals (1 with confirmed cSVD and 1 with an unknown disease status). Both of the *LAMB1* variants identified were interpreted as a variant of uncertain significance (see Data S1 and Figure S3 for full details).

**Table 3:** Fisher Exact Test Comparing the Burden of Rare Variants in Recently Implicated cSVD Genes

All samples					Excluding known cSVD mutations			
Recently implicated cSVD genes	Variant carriers in unrelated cases, n (%)	Variant carriers in unrelated controls, n (%)	Predicted mode of inheritance (posterior probability)	OR, *P* value	Variant carriers in unrelated cases, n (%)	Variant carriers in unrelated controls, n (%)	Predicted mode of inheritance (posterior probability)	OR, *P* value
*ARHGEF15*	2 (1.6)	149 (1.4)	Dominant (0.65)	1.11, 0.54	1 (0.9)	149 (1.4)	Unknown	0.65, 0.78
*ATP11B*	3 (2.4)	105 (1.0)	Dominant (0.91)	2.39, 0.14	1 (0.9)	105 (1.0)	Unknown	0.93, 0.66
*CECR1*	0	63 (0.6)	Unknown	0, 1.00	0	63 (0.6)	Unknown	0, 1.00
*CSF1R*	0	79 (0.8)	Unknown	0, 1.00	0	79 (0.8)	Unknown	0, 1.00
*LAMB1*	3 (2.4)	254 (2.4)	Dominant (0.62)	0.97, 0.60	2 (1.9)	254 (2.4)	Dominant (0.66)	0.77, 0.74
*PLOD3*	3 (2.4)	144 (1.4)	Dominant (1.00)	1.73, 0.26	3 (2.8)	144 (1.4)	Dominant (1.00)	2.07, 0.19

Comparison between unrelated cSVD cases with (n_1_=126) or without a known mutation (n_2_=106) vs unrelated controls (n_3_=10 380). cSVD indicates cerebral small‐vessel disease; and OR, odds ratio.

### Identification of Novel Monogenic cSVD Genes

To enhance the ability to detect rare associations, we conducted a SKAT‐O analysis encompassing >2.1 million rare protein‐truncating and missense variants (MAF<0.001 in unrelated controls) spanning 57 755 genes, excluding previously reported cSVD genes (Figure S3). Three genes showed significant associations (*P*<5×10^−8^) with cSVD: RP4‐568F9.3 (also known as ZNF133‐AS1, adjusted *P*=7.1×10^−25^), RP3‐466I7.1 (adjusted *P*=8.9×10^−16^), and ZNF209P (adjusted *P*=1.0×10^−15^). RP4‐568F9.3, a noncoding antisense RNA expressed in the brain, displayed a rare noncoding variant (ENST00000436848.1: n.1G>A) in 5 related individuals, including 1 male index case confirmed with cSVD. RP3‐466I7.1, expressed in the brain, harbored a splice variant (ENST00000436728.1: n.353+1A>G) identified in 3 subjects, 1 of whom was confirmed with cSVD. ZNF209P, a pseudogene, presented a splice variant (ENST00000593450.1: n.225‐2A>G) found in 2 related subjects, with only the female index case confirmed with cSVD (Figure S5). These variants, although located in noncoding genes, were predicted to affect splice sites or alter noncoding exon sequences within noncoding transcripts.

Last, we examined the suggestive significant genes from SKAT‐O (*P*<1×10^−5^) that encoded key matrisome proteins expressed in the brain (Table 4). Associations were observed on *FAM20C*, *INHA*, *LAMC1*, and *VWA5B2* when we compared the burden of rare variants between unrelated cases and controls. Details about the rare variants in these genes are summarized in Table S3. Those that occurred in multiple affected family members were selected for family‐based analysis and are described in Data S2 and Figures S6 to S9. Limited evidence showed they were pathogenic.

**Table 4:** Fisher Exact Test Comparing the Burden of Rare Variants in the 26 Genes Detected in SKAT‐O Analysis

Genes from SKAT‐O	Variant carriers in unrelated cases, n (%)	Variant carriers in unrelated controls, n (%)	Predicted mode of inheritance (posterior probability)	OR, *P* value
*A2M*	6 (5.7)	257 (2.5)	Dominant (1.00)	2.36, 0.05
*ADAM11*	3 (2.8)	127 (1.2)	Dominant (1.00)	2.35, 0.14
*ADAMTS3*	5 (4.7)	234 (2.3)	Dominant (1.00)	2.15, 0.09
*ANXA6*	5 (4.7)	205 (2.0)	Dominant (1.00)	2.46, 0.06
*BMP1*	5 (4.7)	242 (2.3)	Dominant (1.00)	2.07, 0.11
*COL11A2*	8 (7.5)	434 (4.2)	Dominant (1.00)	1.87, 0.08
*COL21A1*	4 (3.8)	261 (2.5)	Dominant (1.00)	1.52, 0.28
*COL8A2*	2 (1.9)	199 (1.9)	Unknown	0.98, 0.61
*EGLN3*	2 (1.9)	48 (0.5)	Unknown	4.14, 0.09
*FAM20C* *	7 (6.6)	196 (1.9)	Dominant (1.00)	3.67, 0.004
*FGF13*	1 (0.9)	17 (0.2)	Unknown	5.80, 0.17
*FGF2*	3 (2.8)	150 (1.4)	Dominant (1.00)	1.99, 0.20
*HCFC1*	1 (0.9)	171 (1.6)	Unknown	0.57, 0.83
*IL11*	2 (1.9)	56 (0.5)	Unknown	3.54, 0.12
*INHA* *	5 (4.7)	162 (1.6)	Dominant (1.00)	3.12, 0.03
*LAMC1* *	10 (9.4)	412 (4.0)	Dominant (1.00)	2.52, 0.01
*LGALS8*	1 (0.9)	132 (1.3)	Unknown	0.74, 0.74
*PCSK6*	8 (7.5)	399 (3.8)	Dominant (1.00)	2.04, 0.05
*PLXDC1*	5 (4.7)	197 (1.9)	Dominant (1.00)	2.56, 0.05
*RP3‐466I7.1* * ^,^ †	3 (2.8)	0	Unknown	NA
*RP4‐568F9.3* * ^,^ †	2 (1.9)	0	Unknown	NA
*SULF1*	3 (2.8)	157 (1.5)	Dominant (0.72)	1.90, 0.22
*SULF2*	5 (4.7)	244 (2.4)	Dominant (1.00)	2.06, 0.11
*VEGFC*	1 (0.9)	70 (0.7)	Unknown	1.40, 0.52
*VWA5B2* *	9 (8.5)	383 (3.7)	Dominant (1.00)	2.42, 0.02
*ZNF209P* * ^,^ †	1 (0.9)	0	Unknown	NA

Three genome‐wide significant genes (*P*<1×10^−8^) and 23 suggestive significant matrisome‐related genes (*P*<1×10^−5^) were identified in cerebral small‐vessel disease unrelated cases (n_1_=106) vs unrelated controls (n_2_=10 380), excluding known mutations. OR indicates odds ratio; and SKAT‐O, sequence kernel association test with optimal combination of variants.

* Significance at *P*<0.05.

† Denotes genome‐wide significant genes in SKAT‐O.

## Discussion

In this cohort of 257 patients with suspected familial cSVD, and 13 086 controls, who had WGS performed, we first screened for mutations in known genes previously reported to cause monogenic SVD and then applied a variety of bioinformatic techniques to look for novel genes.

A total of 8.9% of the cases carried a mutation in genes previously described to cause cSVD, the most common of which were *NOTCH3* and *HTRA1*. All *HTRA1* cases were heterozygous, and no homozygous cases of cerebral autosomal recessive arteriopathy with subcortical infarcts and leukoencephalopathy were found. We also detected 2 mutations in *COL4A1/2* and a single mutation in *LAMB1*, a recently reported monogenic form of cSVD. Excluding these already described causes of monogenic cSVD, rare predicted deleterious variants in the Genomics England gene panel were found in 23.6% of the unrelated subjects with cSVD, which is similar to the proportion of cases explained by routine clinical genetic testing in another cohort,^11^ but no association was found with cSVD in gene‐based burden tests. Furthermore, SKAT‐O analysis revealed that noncoding genes, such as *RP4‐568F9.3*, *RP3‐466I7.1*, and *ZNF209P*, as well as genes that encode matrisome proteins in the brain, including *FAM20C*, *INHA*, *LAMC1* (*Laminin Subunit Gamma 1*), and *VWA5B2*, may be associated with cSVD. These results extend previous work by identifying previously unexplored genes that may contribute to the development of cSVD, although the associations now need replicating in other cohorts.

*RP4‐568F9.3*, *RP3‐466I7.1*, and *ZNF209P* were the top 3 novel genes most strongly associated with cSVD on SKAT‐O analysis. Although the function of *RP3‐466I7.1* remains unknown, *RP4‐568F9.3* and *ZNF209P* appeared to be related to zinc transcription factor (ZNF) proteins, ZNF133 and ZNF208, respectively. Other ZNF proteins have been found to regulate cellular processes underlying the development and differentiation of various tissues. Notably, a sequence similarity (83.1%) was observed between the putative coding portions of *ZNF209P* and *ZNF208*,^28^ which were associated with ischemic stroke.^29^, ^30^ This suggests that although *RP4‐568F9.3* and *ZNF209P* did not encode functional proteins, their gene products may influence expression of complementary functional gene sequences that may be involved in cSVD. Nonetheless, this remains speculative and needs further functional investigation.

The matrisome proteins expressed in the brain were prioritized in our SKAT‐O analysis given their likely involvement in cSVD development.^9^, ^10^ We found that rare variants in *FAM20C*, *INHA*, *LAMC1*, and *VWA5B2* were overrepresented in cSVD cases. The proteins encoded by these genes were not known to cause cSVD but may interact with proteins implicated in cSVD. For example, LAMC1 binds with LAMB1 and other laminin monomers expressed in the brain to form different types of laminin proteins, which can be disrupted in cSVD.^31^, ^32^ However, further studies are needed to replicate our findings in different cohorts and establish the functional consequences of these variants in relation to cSVD.

Our study has several strengths. First, it is 1 of the largest cohorts with well‐phenotyped suspected familial cSVD and was accompanied by a large control cohort for comparison. We applied a variety of different bioinformatic approaches. However, it also has limitations. First, only a few cSVD cases were found with a rare deleterious variant, which restricted the power to detect genes associated with the disease. These findings need to be replicated in further independent data sets to confirm their validity. The limited number of family members in many cases made it difficult to assess whether variants segregate with disease. Additional family members with MRI would allow more certain identification of potential genes on segregation analysis. As shown in the SKAT‐O analysis, many genes had *P* values of ≈1×10^−5^ (Figure S4), which may be false positives because of systematic error associated with the lack of cases. A larger overall sample size would improve the power to detect associations with novel cSVD genes. Second, the non‐cSVD group used in this study consisted of NBR study participants with other rare diseases. Although this should not reduce our ability to detect overrepresentation of cSVD‐specific rare variants, it could limit detection if variants in a gene cause multiple phenotypes by affecting different protein domains. To address this, additional analysis of variant function and distribution within genes, as well as detailed phenotypic information about the non‐cSVD group, is necessary. Third, the genetic architecture of cSVD may be more complex than previously thought and could involve a combination of rare and common variants, both in protein‐coding and noncoding regions of the genome.^33^ Although this study only focused on rare variants that were more likely to have a large effect on disease risk or severity, common variants may also contribute to the overall genetic risk of developing cSVD.^34^, ^35^ Additionally, noncoding variants, which do not directly affect the amino acid sequence of a protein, may regulate gene expression and contribute to the development of cSVD. Hence, the potential effects of common and noncoding variants on cSVD require further investigation.

The most common variants in genes already described to cause cSVD were *NOTCH3*, causing CADASIL (ie, variants altering a cysteine residue in an epidermal growth factor‐like repeat of NOTCH3, n=11), *HTRA1* (n=9), and *COL4A2* (n=2).^2^ In addition, a recently reported pathogenic variant in *LAMB1* ^8^ was identified. Together, these mutations accounted for 8.9% of all cases, of which all were heterozygous (Table 1).

In conclusion, in a cohort of familial WGS, we identified mutations in 4 already identified genes (*NOTCH3*, *HTRA1*, *COL4A1/2*, and *LAMB1*). Moreover, predicted deleterious variants in previously established genes were present in approximately a quarter of unrelated cases with monogenic cSVD, but none of the genes were associated with the disease. Additionally, rare sequence variants in other genes, including *FAM20C*, *INHA*, *LAMC1*, *RP3‐466I7.1*, *RP4‐568F9.3*, *VWA5B2*, and *ZNF209P*, could potentially contribute to the development of cSVD. These genes may play a role in tissue development and maintaining the function of brain endothelial cells.

## Sources of Funding

This research was funded by a British Heart Foundation (BHF) program grant (RG/F/22/110052). Dr Cho is supported by a PhD studentship awarded by the Cambridge BHF Centre of Research Excellence. Infrastructural support was provided by the Cambridge British Heart Foundation Centre of Research Excellence (RE/18/1/34212), and Cambridge University Hospitals National Institute for Health Research (NIHR) Biomedical Research Centre (BRC‐1215‐20 014). The views expressed in this publication are those of the authors and not necessarily those of the NIHR, National Health Service, or UK Department of Health and Social Care.

## Disclosures

None.

## Supporting information

Data S1–S2
Tables S1–S3
Figures S1–S9
