# Supplementary material for: Rare Sequence Variation Underlying Suspected Familial Cerebral Small‐Vessel Disease
Source: J Am Heart Assoc. 2024 Jul 31;13(15):e035771. doi: 10.1161/JAHA.123.035771 (PMC11964016; doi:10.1161/JAHA.123.035771)
Supplement: Supplementary file 1 — Data S1–S2 Tables S1–S3 Figures S1–S9 [file JAH3-13-e035771-s001.pdf]

Supplemental Material

Supplemental Methods

Data S1.

<u>Carriers of *LAMB1*:p.Pro975Ser variant</u>

The ENSP00000222399.6:p.Pro975Ser variant in *LAMB1* exon 22 was carried by an affected individual (proband, 08-027) and her son (08-035, **Figure S3**). The proband first suffered from migraine with and without aura at age 13, and was then diagnosed with depression and cognitive impairment at ages 30 and 57, respectively. Neuroimaging around the age of 58 detected confluent WMH, suggestive of moderate to severe ischaemic changes which led to a suspicion of CSVD. Both the proband and her son, as well as two other family members, had symptoms of Cowden's syndrome, which is a genetic disorder characterized by multiple noncancerous, tumour-like nodules (not known to be associated with cSVD). Nonetheless, it was uncertain whether the recruited son (08-035), who was clinically depressed, had cSVD as neuroimaging data was unavailable. Although the p.Pro975Ser variant was rare, with an allele frequency of less than 1 in 10,000 in the GnomAD control database (rs1283431054), it was found in four other unaffected individuals in the NBR-RD cohort who were diagnosed with neurodevelopmental disorder, multiple primary tumours and Leber hereditary optic neuropathy. Based on the evidence above, the p.Pro975Ser variant was interpreted as a VUS.

<u>Carrier of *LAMB1*:p.Asp611Asn variant</u>

Lastly, a male proband of a singleton family carried a ENSP00000222399.6:p.Asp611Asn variant in *LAMB1* exon 15. He started to have focal motor seizures and cognitive deficits from around age 38 and experienced a stroke-like episode at age 39. MRI detected abnormal white matter changes around ventricles, right superior frontal gyrus and left anterior temporal pole. There was no sign of microbleeds. The variant (rs146589212) was previously reported in the GnomAD database with a frequency of 3 in 140,058. However, due to the lack of segregation and functional data, this variant was interpreted as a VUS.

Data S2

<u>Carrier of *FAM20C*:p.Val552Ile variant</u>

A missense variant in *FAM20C* (ENSP00000322323.5:p.Val552Ile) was found in an index case and her two daughters (**Figure S6**). The index case suffered from encephalopathy at age 55. Brain MRI showed white matter abnormalities but no typical features of CADASIL. However, the disease status of her two daughters, who also carried the same variant, was unknown and no MRI was available. Additionally, the p.Val552Ile variant was found in three other participants in the control group but was absent from population databases.

<u>Carrier of *INHA*:p.His218Tyr variant</u>

Most of the rare variants found in *INHA* were upstream gene variants, only one was missense (**Table S3**). The latter (ENSP00000243786.2:p.His218Tyr) was carried by five members of a family. The proband, his mother and maternal uncle were affected,

while the status of his sister and brother was unknown. The proband suffered from migraine with aura at age 54, his mother had stroke at age 73, and his maternal uncle had haemorrhagic stroke at age 68. Brain MRI of the proband showed periventricular WMH due to cortical atrophy (**Figure S7)**. This *INHA* variant was found in the proband's brother and sister (with unknown status) as well as a control subject but was absent from population databases.

Carrier of *VWA5B2*:p.Thr819Met variant

A missense variant in *VWA5B2* (ENSP00000398688.2:p.Thr819Met) was found in two related patients and was absent from our control group and population databases. It was carried by an index case and her brother who did not have MRI to confirm his disease status. The index had her first stroke at age 56 and her MRI showed extensive features of cSVD and numerous lacunar infarcts (**Figure S8**).

Carrier of *LAMC1*:p.Leu1520Phe variant

A rare variant in *LAMC1* (ENSP00000258341.3:p.Leu1520Phe) was identified in a pair of affected mother and daughter (**Figure S9**). The index case of this family (05-003) was the sister of the affected mother (05-004) but did not have the variant. The affected daughter (i.e., the proband's niece, 05-005) also carried another rare *LAMC1* variant (p.Ala1416Val) which was not found in her mother or aunt. The affected mother had migraine, depression, anxiety, cognitive impairment and sensory problems. Her brain MRI showed extensive WMH and mild-to-moderate atrophy. Her daughter was diagnosed with depression at age 16 and had a haemorrhagic stroke at age 43. *LAMC1*:p. Leu1520Phe variant was extremely rare in the general population – 1/140,138 in GnomAD and 2/264,690 in TOPMED. Variants in this gene were not reported to be associated with cSVD or stroke.

Table S1 Missense and protein-truncating variants in typical cSVD genes identified in cSVD cases. cSVD, Cerebral Small Vessel Disease.

Gene	Variant	HGVS (protein)	Exon	Consequence type	dbSNP ID
ABCC6	16:16263524_C/T	p.Gly992Arg	22 of 31	missense variant	rs72657692
ABCC6	16:16306070_A/G	p.Ser212Pro	6 of 31	missense variant	
ABCC6	16:16306091_C/A	p.Glu205Ter	6 of 31	stop gained	
ABCC6	16:16308218_T/G	p.Asp188Ala	5 of 31	missense variant	
ABCC6	16:16313504_T/A	p.Lys127Asn	4 of 31	missense variant	
ABCC6	16:16313741_G/A	p.Gln95Ter	3 of 31	stop gained	
ABCC6	16:16315622_C/G	p.Ala35Pro	2 of 31	missense variant	
ABCC6	16:16317287_G/T	p.Ala2Asp	1 of 31	missense variant	
APP	21:27347439_G/A	p.Arg468Cys	11 of 18	missense variant	
CACNA1A	19:13318774_T/A	p.Thr2292Ser	47 of 47	missense variant	
CACNA1A	19:13320252_G/A	p.Arg2134Cys	45 of 47	missense variant	rs121908235
CACNA1A	19:13323491_C/T	p.Gly2002Ser	41 of 47	missense variant	
COL3A1	2:189850466_C/T	p.Pro137Ser	4 of 51	missense variant	
COL3A1	2:189872295_C/G	p.Arg1109Gly	45 of 51	missense variant	rs112371422
COL4A1	13:110807651_C/G	p.Trp1578Cys	50 of 52	missense variant	
COL4A2	13:111009840_C/T	p.Pro41Ser	4 of 48	missense variant	rs540581830
COL4A2	13:111117807_C/T	p.Thr611Met	25 of 48	missense variant	rs373521529
COL4A2	13:111164386_G/A	p.Gly1663Ser	48 of 48	missense variant	rs12877501
COLGALT1	19:17670163_C/T	p.Arg102Trp	2 of 12	missense variant	rs377086776
GLA	X:100652972_A/G	p.Leu372Pro	7 of 7	missense variant	

Table S2 Missense and protein-truncating variants in recently implicated cSVD genes identified in cSVD cases. cSVD, Cerebral Small Vessel Disease.

Gene	Variant	HGVS (protein)	Exon	Consequence type	dbSNP ID
ARHGEF15	17:8215614_A/C	p.Asp86Ala	2 of 16	missense variant	
ATP11B	3:182631763_A/G	p.Ser1145Gly	29 of 30	missense variant	rs752004427
LAMB1	7:107594131_G/A	p.Pro975Ser	22 of 34	missense variant	
LAMB1	7:107605100_C/T	p.Arg532Gln	14 of 34	missense variant	rs143390327
PLOD3	7:100850143_C/T	p.Glu660Lys	18 of 19	missense variant	rs761576023
PLOD3	7:100854987_C/T	p.Ala415Thr	12 of 19	missense variant	rs144508814
PLOD3	7:100859699_A/C	p.Phe110Leu	3 of 19	missense variant	rs551969292

Table S3 Rare variants in genes with genome-wide significant (SKAT-O $P<1\times10^{-8}$) and suggestive significant (SKAT-O $P<1\times10^{-5}$) associations with cSVD and that were present in cSVD cases. SKAT-O, Sequence Kernel Association Test with Optimal combination of variants; cSVD, Cerebral Small Vessel Disease.

Gene	Variant	HGVS (protein)	Exon	Consequence type	dbSNP ID
ADAM11	17:42849767_G/A	p.Gly204Asp	8 of 27	missense variant & splice region variant	
ADAM11	17:42849808_C/T	p.Arg218Trp	8 of 27	missense variant	rs544346894
ADAM11	17:42850401_C/T	p.Ser258Leu	10 of 27	missense variant	rs764055319
FAM20C	7:193254_G/T	p.Val19Leu	1 of 10	missense variant	rs529612835
FAM20C	7:193384_A/C	p.Gln62Pro	1 of 10	missense variant	
FAM20C	7:195681_G/A	p.Ala245Thr	2 of 10	missense variant	rs61730252
FAM20C	7:288383_CTTCATCTCTCCAGGTAGCCTGGCACGGGGGCCCGCA/C		5 of 10	splice donor variant & coding sequence variant & intron variant	
FAM20C	7:299767_G/A	p.Ala526Thr	10 of 10	missense variant	
FAM20C	7:299845_G/A	p.Val552Ile	10 of 10	missense variant	rs753843872
FAM20C	7:299939_C/T	p.Ala583Val	10 of 10	missense variant	rs557222235
INHA	2:220434973_T/C			upstream gene variant	rs759218624
INHA	2:220435035_A/G			upstream gene variant	
INHA	2:220435063_G/A			upstream gene variant	rs367952566
INHA	2:220435746_A/G			upstream gene variant	
INHA	2:220439799_C/T	p.His218Tyr	2 of 2	missense variant	

LAMC1	1:182992867_C/T	p.Arg6Trp	1 of 28	missense variant	
LAMC1	1:183072665_T/G	p.Asp207Glu	2 of 28	missense variant	rs201591027
LAMC1	1:183079691_A/G	p.Lys308Arg	4 of 28	missense variant	rs139092535
LAMC1	1:183079729_C/T	p.Pro321Ser	4 of 28	missense variant	rs142614579
LAMC1	1:183086486_T/G	p.Asp532Glu	9 of 28	missense variant	rs61752348
LAMC1	1:183091040_G/A	p.Ala725Thr	12 of 28	missense variant	rs147401305
LAMC1	1:183105593_A/G	p.Gln1396Arg	25 of 28	missense variant	rs748236662
LAMC1	1:183105653_C/T	p.Ala1416Val	25 of 28	missense variant	rs148395845
LAMC1	1:183109623_C/T	p.Leu1520Phe	27 of 28	missense variant	rs766398357
LAMC1	1:183111678_A/G	p.Asp1528Gly	28 of 28	missense variant	rs751829591
RP3-466I7.1	6:108709069_A/G			splice donor variant & noncoding transcript variant	
RP4-568F9.3	20:18295755_C/TRUE		1 of 2	Non-coding transcript exon variant	rs151186857
VWA5B2	3:183951125_C/A	p.Thr157Asn	3 of 19	missense variant	rs548730521
VWA5B2	3:183951465_C/T	p.Pro211Leu	4 of 19	missense variant	rs571169902
VWA5B2	3:183952090_C/T	p.Ala242Val	5 of 19	missense variant	
VWA5B2	3:183957540_C/T	p.Thr819Met	15 of 19	missense variant	rs772459643
VWA5B2	3:183957585_C/T	p.Pro834Leu	15 of 19	missense variant	rs201206346
VWA5B2	3:183958603_G/C	p.Arg922Pro	16 of 19	missense variant	
VWA5B2	3:183958606_C/G	p.Ala923Gly	16 of 19	missense variant	
VWA5B2	3:183959483_C/A	p.Ser1129Tyr	19 of 19	missense variant	

ZNF209P	19:22653757_A/G			splice acceptor variant & noncoding transcript variant	

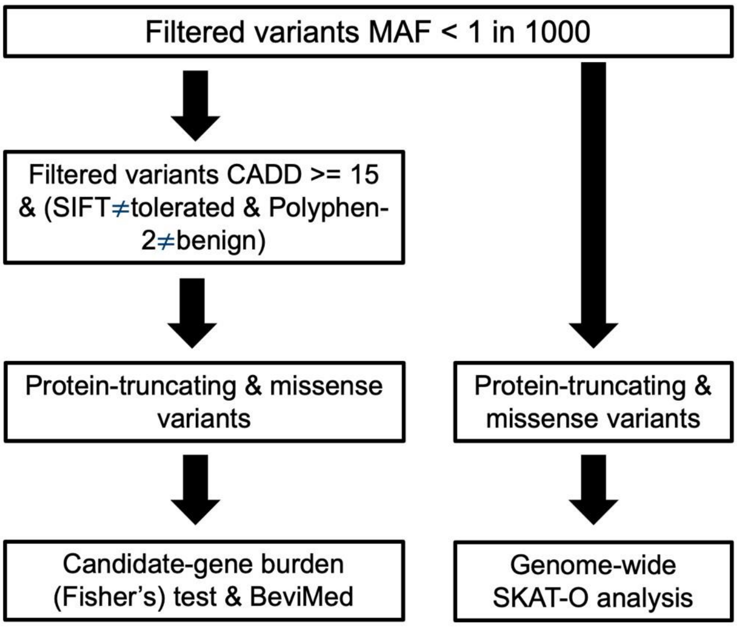

Figure S1 Analysis strategies to assess the involvement of rare variants in candidate cSVD genes by burden test, BeviMed, and SKAT-O.

Abbreviations: BeviMed, Bayesian Evaluation of Variant Involvement in Mendelian Disease; CADD, Combined Annotation Dependent Depletion; MAF, Minor Allele Frequency; SKAT-O, Sequence Kernel Association Test with optimal combination of variants; SIFT, Sorting Intolerant From Tolerant

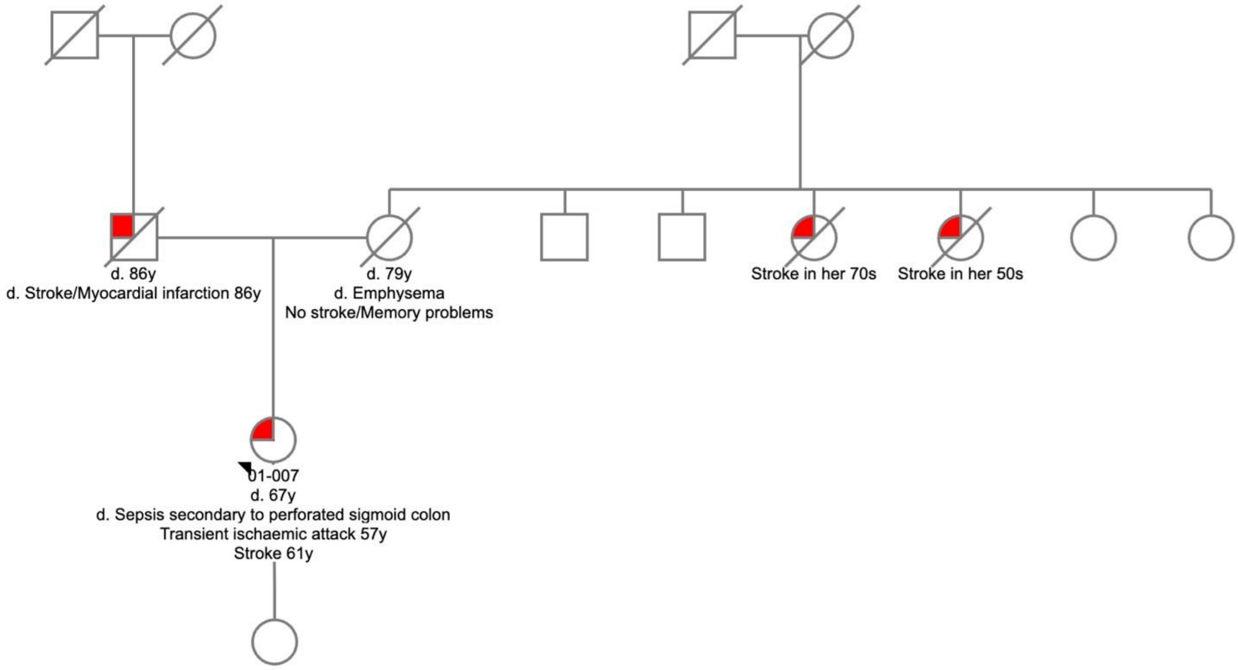

Figure S2 A singleton family of a *LAMB1*:p.Leu1730Ter variant carrier. Red represents cSVD. cSVD, Cerebral Small Vessel Disease.

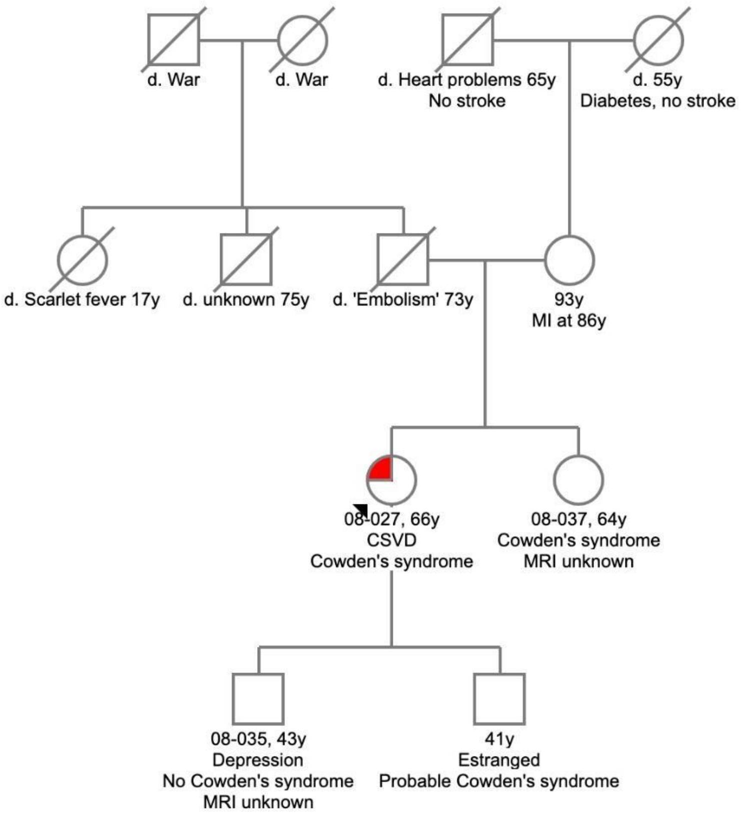

Figure S3 Pedigree of F105, a family of three participants with WGS data. The proband with CSVD and her son with unknown status both carried LAMB1:p.Pro975Ser. The maternal aunt with unknown status did not carry this variant. Red represents cSVD.

WGS, Whole Genome Sequencing; cSVD. Cerebral Small Vessel Disease.

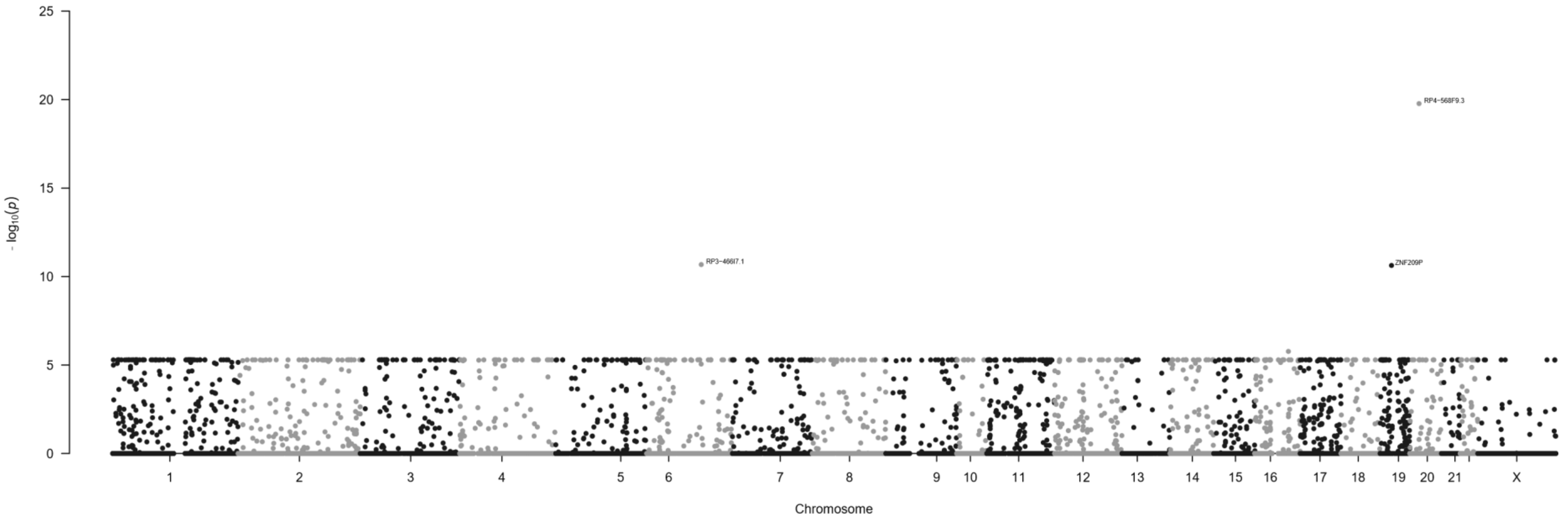

Figure S4 Manhattan plot of SKAT-O analysis of rare protein-truncating and missense variants (excluding cases with a known mutation). Filtered variants (MAF < 0.001 in the unrelated controls) were grouped per gene and were tested for overrepresentation in cSVD cases versus control. The negative decadic log of Bonferroni-adjusted *P*-values were plotted against the chromosomal location of each gene. SKAT-O, Sequence Kernel Association Test with Optimal combination of variants.

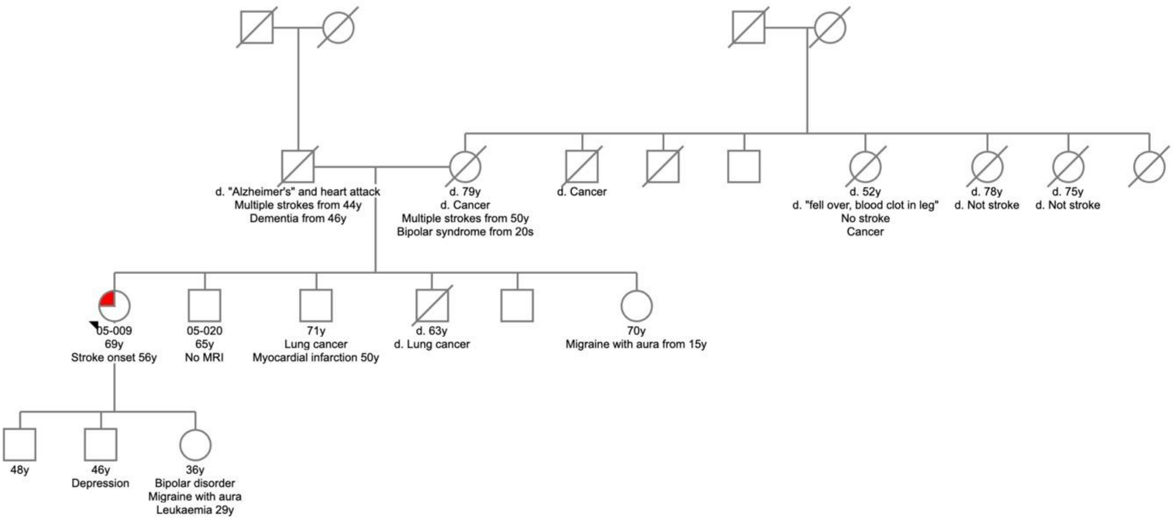

Figure S5 A family of a sister (proband) and brother carrying a splice variant in *ZNF209P*. Red represents cSVD. cSVD, Cerebral Small Vessel Disease.

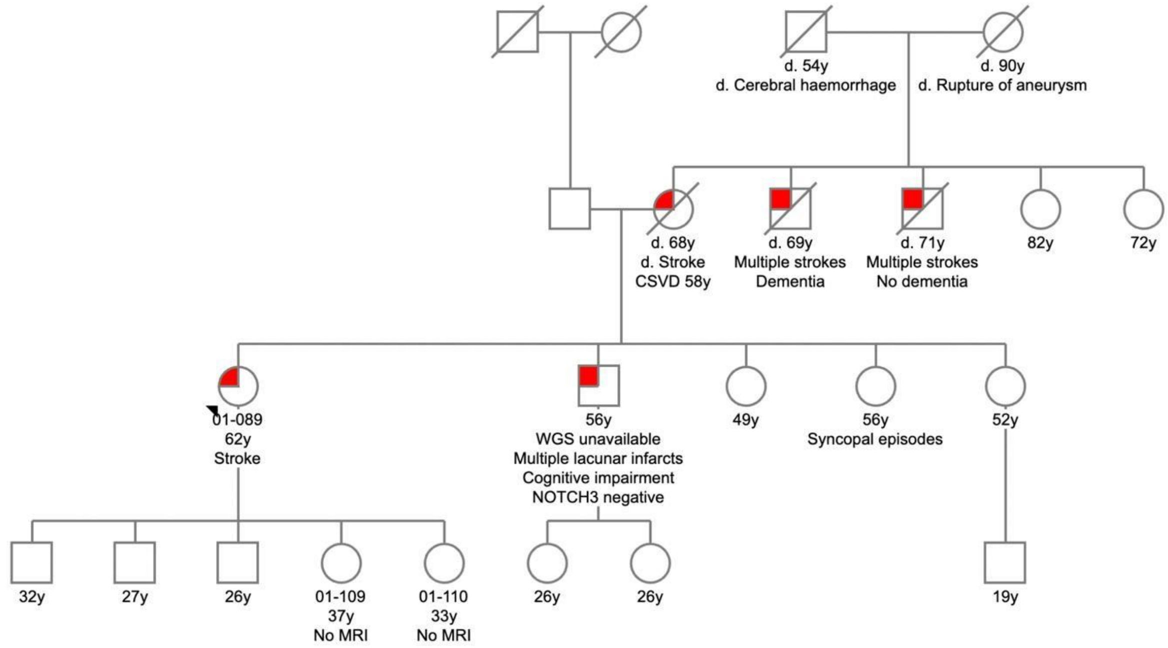

Figure S6 Pedigree of F108, a family of a mother (proband, 01-089) and her two daughters (01-109 and 01-110) carrying a missense variant in *FAM20C* (p.Val552Ile).
Red represents cSVD. cSVD, Cerebral Small Vessel Disease.

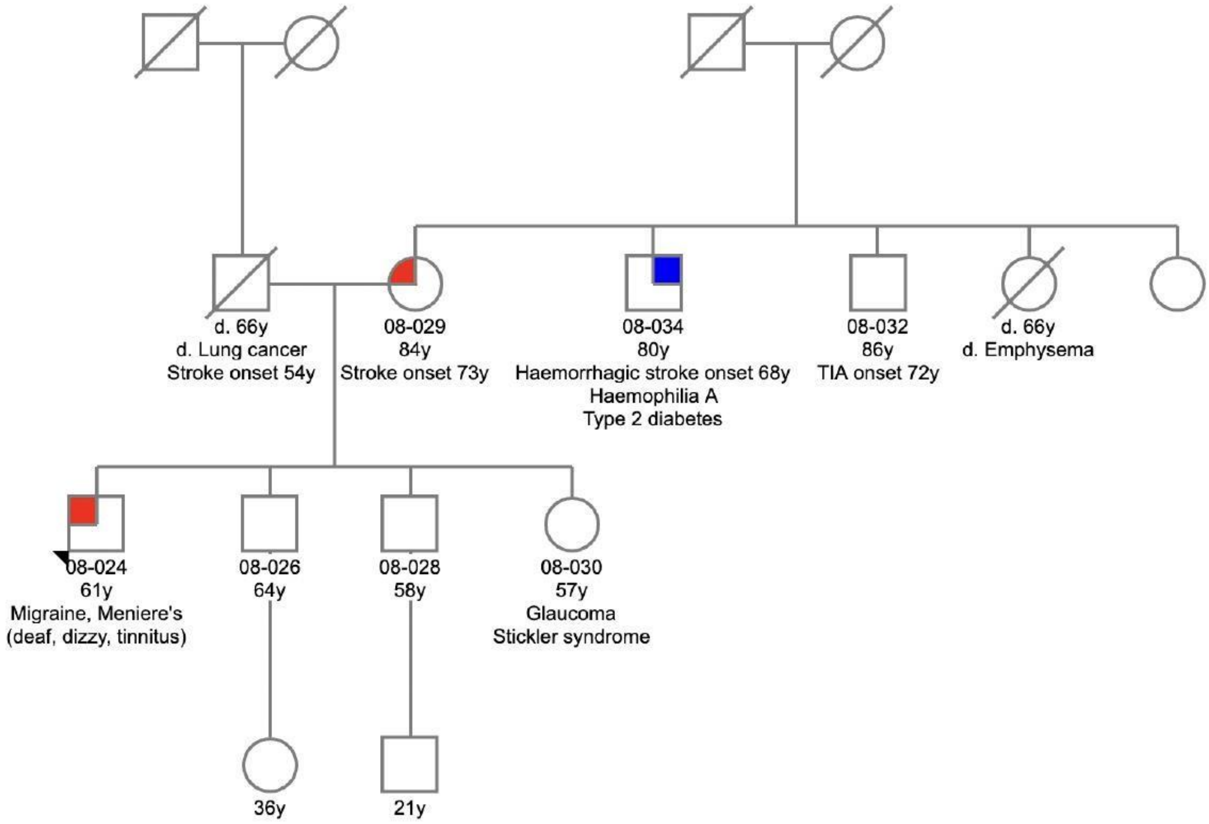

Figure S7 Pedigree of F89, a family of seven recruited subjects, with the proband, his affected mother, three siblings, and two maternal uncles. Only subjects 08-024, 08028, 08-029, 08-030 and 08-034 carried the missense variant in *INHA* (p.His218Tyr). Red represents cSVD; Blue represents haemorrhagic stroke. cSVD, Cerebral Small Vessel Disease.

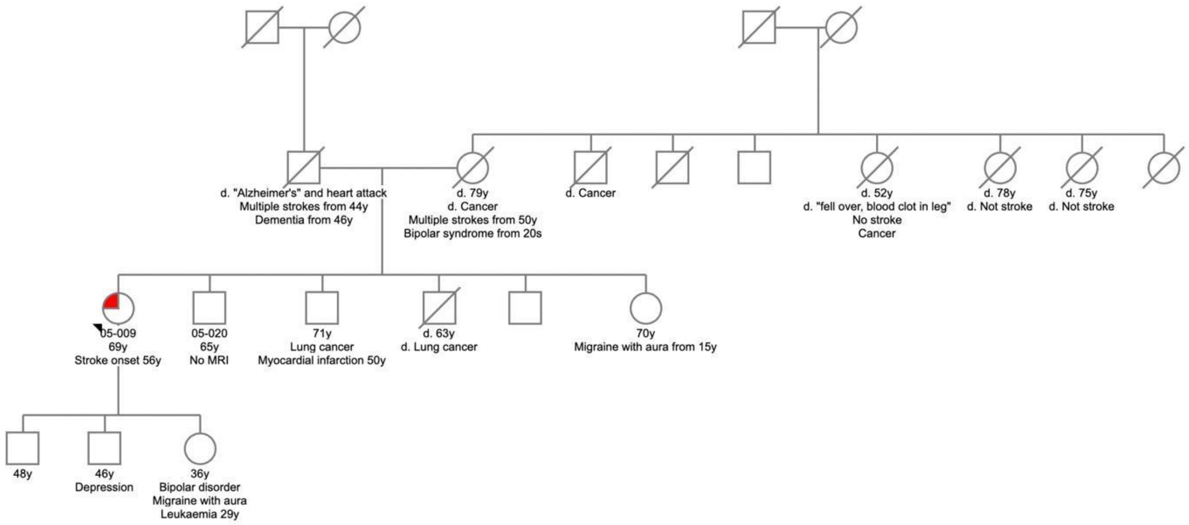

Figure S8 Pedigree of F93, a family of two recruited subjects, with the proband and her brother, who both carried the missense variant in *VWA5B2* (p.Thr819Met). Red represents CSVD.

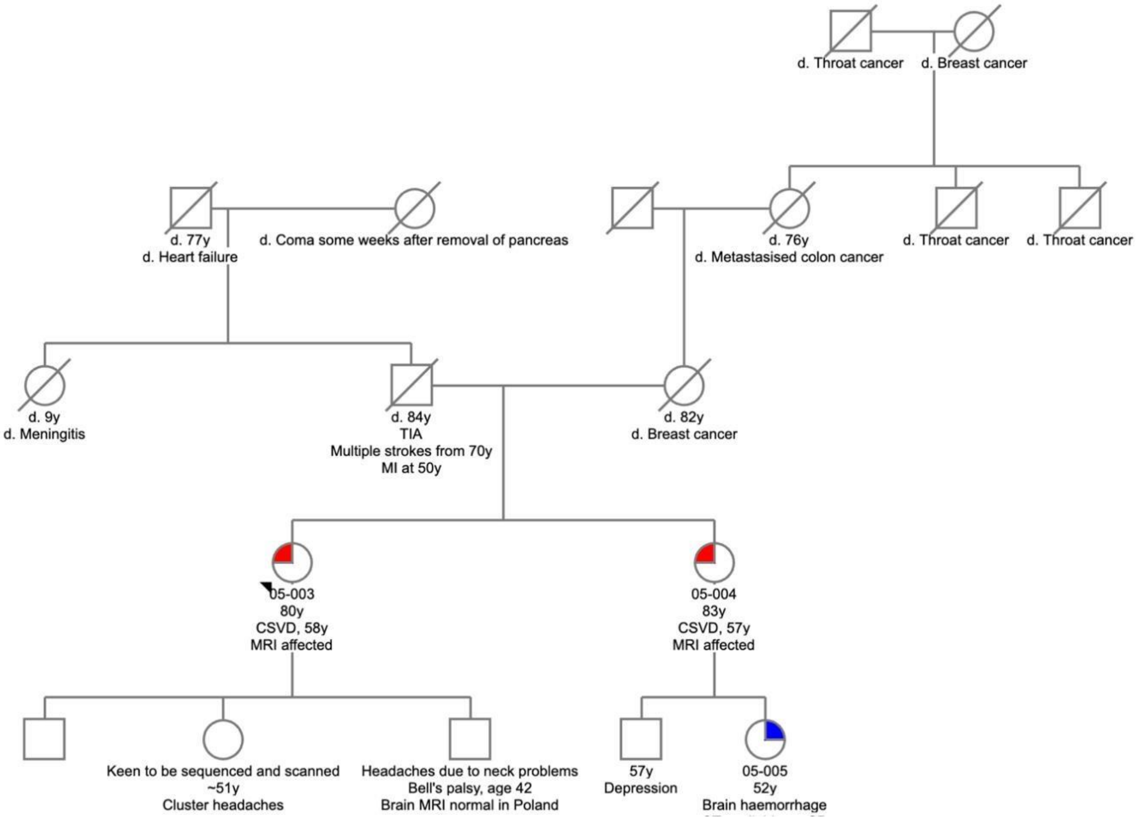

Figure S9 Pedigree of F07, a family of an index case (05-003), her sister (05-004) and niece (05-005). 05-004 and 05-005 both carried a *LAMC1*:p.Leu1520Phe variant. Red represents affected individuals with cSVD; Blue represents affected individuals with haemorrhagic stroke. cSVD, Cerebral Small Vessel Disease.
